# Enhancing prediction of inpatient deterioration by combining clinical and nurse concern features, with or without temporal clustering

**DOI:** 10.1093/jamiaopen/ooag077

**Published:** 2026-05-20

**Authors:** Yik-Ki Jacob Wan, Samir E Abdelrahman, Julio C Facelli, Karl Madaras-Kelly, Kensaku Kawamoto, Deniz Dishman, Kenrick Cato, Sarah C Rossetti, Guilherme Del Fiol

**Affiliations:** Department of Biomedical Informatics, University of Utah, Salt Lake City, UT 84108, United States; Department of Biomedical Informatics, University of Utah, Salt Lake City, UT 84108, United States; Department of Biomedical Informatics, University of Utah, Salt Lake City, UT 84108, United States; College of Pharmacy, Idaho State University, Meridian, ID 83642, United States; Department of Biomedical Informatics, University of Utah, Salt Lake City, UT 84108, United States; Cizik School of Nursing Department of Research, University of Texas Health Science Center at Houston, Houston, TX 77030, United States; Children’s Hospital of Philadelphia, Philadelphia, PA 19104, United States; Biomedical Informatics and Nursing, Columbia University, New York, NY 10032, United States; Department of Biomedical Informatics, University of Utah, Salt Lake City, UT 84108, United States

**Keywords:** early warning score, machine learning, predictive modeling, nursing assessment, clinical intuition

## Abstract

**Background:**

Early warning systems (EWSs) help clinicians identify deteriorating patients using clinical data, such as vital signs. However, standard systems struggle to capture nuanced nursing concerns. The Healthcare Process Model-ExpertSignals (HPM-ExpertSignals) framework describes how nurses’ concerns are reflected in their documentation patterns. While a recent trial showed positive outcomes, the predictive gain of combining both data types remains unquantified.

**Objectives:**

We evaluated improvements in F-measure by combining HPM-ExpertSignals with clinical data using the k-shape temporal clustering algorithm.

**Materials and Methods:**

Six models were compared based on their features and the inclusion of k-shape. Models were trained to predict patient deterioration (cardiac arrest and death) 12 h before the event using a large dataset. The primary outcome was the harmonic mean of precision and recall (F-measure).

**Results:**

The F-measure achieved by the model that uses both feature types was 0.25 (±0.01). The clinical features-only model was 0.16 (±0.01), and the HPM-ExpertSignals-only model was 0.19 (±0.02). F-measures for their corresponding k-Shape models were all at 0.06 (±0.0).

**Discussion:**

The combined model has the highest F-measure among the clinical-only and HPM-ExpertSignals-only models. The low performance of the k-Shape models suggests that k-Shape is not well suited to capturing the specific temporal patterns present in this problem set.

**Conclusion:**

Early warning systems leveraged both clinical data and HPM-ExpertSignals predictors, which may offer clinically significant improvements. Future research should explore alternative temporal pattern algorithms to further refine predictive accuracy.

## Introduction

Identifying patients at risk of deterioration enables clinicians to make care decisions, such as increased monitoring and treatment.^1^ Tools that help clinicians recognize, react, and respond to these at-risk patients often include early warning systems (EWSs) driven by computational models designed to capture the patient’s state.^2^^,^^3^ Although many advanced machine learning algorithms have emerged in the research landscape, basic scoring models, such as modified early warning score (MEWS), remain prevalent in clinical settings.^4^

Despite the increased adoption of prediction models, barriers to care escalation persist beyond recognizing at-risk patients.^5^ The barriers the nurses face include challenges in articulating, communicating, or justifying heightened concerns based on subtle indicators of changes in a patient condition, particularly when those subtle indicators are not reflected in vital sign changes or a prediction model has not reached its triggering threshold.^5–7^ Recognizing these obstacles, the Communicating Narrative Concerns Entered by RNs (CONCERN) research team proposed that clinician behaviors, reflected in patterns of interaction with clinical information systems (CIS), are valuable features in predicting patient deterioration as formalized in the Healthcare Process Model-ExpertSignals (HPM-ExpertSignals) framework.^8^ HPM-ExpertSignals outlines a 3-step process: “(1) identify patterns of clinical behaviors from user interaction with CIS; (2) interpret patterns as proxies of an individual’s decisions, knowledge, and expertise; and (3) use patterns in predictive models for associations with outcomes.”^9^ The CONCERN team operationalized this framework by (1) identifying nurses’ CIS interaction behavior patterns observable in the flowsheet timestamps, (2) interpreting these patterns as reflections of nurses’ levels of concern for their patients, and (3) operationalizing the timestamps through the sum of documentation frequency in 24-h windows.^9^^,^^10^ They implemented an information-rich multipatient dashboard called CONCERN EWS that included visual features driven partly by HPM-ExpertSignals data. In a clustered multisite randomized controlled trial, intervention units had 35.6% lower risk of mortality and 11.2% shorter length of stay than usual care units.^11^ In a previous study, we also found HPM-ExpertSignals models performed well even when the training data were sourced from a wide range of hospitals in the United States, suggesting that the approach is generalizable across different hospitals.^12^

Previously, the CONCERN team also identified a potential adoption challenge for the models based on HPM-ExpertSignals.^9^ Since most of the predictors used in current patient deterioration models are based on clinical data, a new class of models solely based on clinicians’ behaviors without clinical data may present a cognitive and acceptance challenge to the end users.^9^ The CONCERN team initially explored the potential value of HPM-ExpertSignals features by combining 3 behavioral features with MEWS using a simple scoring rule. The results were comparable to MEWS but showed potential for earlier triggering.^10^ In a subsequent study, the team expanded the number of HPM-ExpertSignals features and applied various ML algorithms without incorporating clinical data. They found logistic regression (LR) to be the most effective algorithm.^13^ While factors attributed to CONCERN EWS’s success may include implementation, training, visual display design, and prediction algorithm, the effect on model performance (F-measure, precision, recall, etc.) by integrating clinicians’ behaviors and clinical data into a single machine learning model has yet to be investigated. Quantifying the benefits may inform future implementers of the potential gains of other bespoke systems by incorporating this data type alone.

Another challenge is the method of operationalizing the temporal patterns as features. Subject matter experts involved in designing HPM-ExpertSignals emphasized the importance of data temporality.^9^ However, the CONCERN team found no significant improvement in model performance when incorporating temporal patterns using techniques such as abstracting the time of day of observations or employing deep learning to analyze changes in observation frequency.^13^ One inherent difficulty in reasoning with temporal clinical and behavioral data is the variability between event sequences.^14^ Temporal clustering techniques, such as k-Shape, that use dynamic sequence matching may help identify pattern clusters despite significant variability in sequence gaps.^15^

This study addresses the following questions: (1) Given an advanced lead time of 12-36 h before adverse events or discharge, does the model’s harmonic mean of precision and recall (F-measure) improve when combining HPM-ExpertSignals with clinical data vs HPM-ExpertSignals or clinical data alone? (2) Does the observational pattern have a distinct “shape” that can be detected by temporal clustering algorithms such as k-Shape? If so, would the use of k-Shape further improve model performance?

## Materials and methods

### Overview

This study compared 6 different models by varying 2 factors: feature types and temporal reasoning approach (Table 1). First, we compared model performances using different feature types. A clinical data model and an HPM-ExpertSignals model were compared with one that used both. Given its broad adoption in clinical settings, we used the components of MEWS for clinical features.^4^^,^^16^ For HPM-ExpertSignals features, we used nurses’ observation frequency.^13^ Then we explored the benefits of temporal clustering. Using the 3 feature combinations described earlier, we developed 3 new k-Shape models and compared their performance to the corresponding original models. Our hypotheses are: (1) models with both (hybrid) features would outperform the 2 models with only 1 feature type, regardless of temporal clustering and (2) models using k-Shape would outperform models without k-Shape. The primary outcome for all hypotheses was the harmonic means of precision and recall (F-measure). Secondary outcomes included sensitivity, positive predictive value (PPV), area under the receiver-operating characteristic curve, and area under the precision-recall curve (AUPRC). The source code is available in Supplementary Material S1).

**Table 1.** Feature types and temporal clustering approaches used across 6 models compared in the study.

Research design		Features		
		MEWS	HPM-ExpertSignals	Both
Temporal Clustering	Without *k*-Shape	**MEWS only** MEWS > 4	**HPM-ExpertSignals only** Frequency counts + Logistic regression	**Hybrid-LR** MEWS and Frequency counts + Logistic regression
	With *k*-Shape	**MEWS with k-Shape** Hourly component MEWS clustered with k-Shape	**HPM-ExpertSignals** **with k-Shape** Hourly observation totals clustered with k-Shape	**Hybrid with k-Shape** Hourly MEWS Components and observation totals clustered with k-Shape

Abbreviations: HPM-ExpertSignals, Healthcare Process Model-ExpertSignals; MEWS, modified early warning score.

#### Dataset

This study utilized eICU-CRD, a deidentified dataset comprising medical records from intensive care unit (ICU) admissions at 208 US hospitals using the Philips eICU system.^17^ The dataset includes vital signs, medication administration records, and flowsheet entries. While actual timestamps were removed as a part of the deidentification process, each observation contains a time variable indicating the number of minutes since admission. As such, absolute variables such as time of day or day of week, which are included in the CONCERN EWS, were not available to be used as features.

### Study population

**Figure 1.**
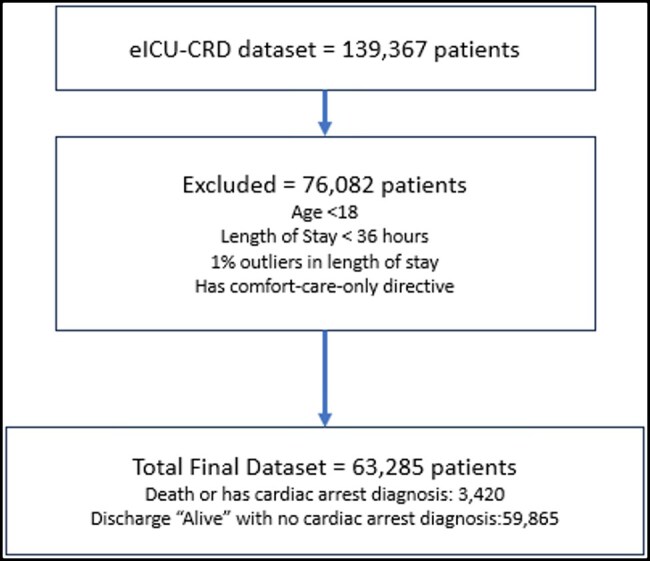
Study population.

We included patients aged 18 years or older whose ICU stay exceeded 36 h to ensure sufficient temporal data for pattern abstraction (Figure 1). Patients receiving comfort care were excluded. Additionally, patients with lengths of stay in the top 1% were excluded as outliers. Using the same approach as in our previous study,^12^ only the first ICU admission for each patient was included to prevent data leakage from correlated training and testing datasets.^18^ To standardize the temporal data window across patients, the dataset only include interval between 36 and 12 h prior to death, discharge alive, or cardiac arrest, regardless of each patient’s total length of stay.^13^

### Patient deterioration labels

Patient deterioration was defined as patients who died or experienced cardiac arrest during their stay (positive cases). Death was identified by a discharge disposition of “EXPIRED,” while cardiac arrest events were determined using diagnosis codes based on logic provided by the eICU-CRD developers.^19^ Conversely, patients discharged alive without experiencing cardiac arrest were labeled as negative cases.

### Features

#### Clinical features

Clinical data features included the components of MEWS: systolic blood pressure, heart rate, respiratory rate, temperature, and level of consciousness. Following MEWS calculation guidelines, each clinical variable was converted into an integer score based on predefined ranges (eg, a heart rate between 111 and 129 beats per minute is assigned +2 points).^20^ The integer points of all features were summed to calculate the total MEWS score. A MEWS score of 3-4 indicates intermediate risk, while a score above 4 indicates high risk.^21^ The threshold scores may vary depending on the desired balance between sensitivity and false positive rates.^22^^,^^23^ If no observation values were available for a given patient hour, a score of zero was assigned for the missing feature. This mimics the simple rule-based heuristic nurses often use, that is, adding the scores up without imputing values.^24^

#### HPM-ExpertSignals features

HPM-ExpertSignals features consisted of documentation frequencies of nursing observations. Observation timestamps were identified using the offset minutes provided in the dataset. Relevant observation types were selected based on expert opinions from previous studies, and the frequencies were represented using the temporal reasoning approaches outlined below.^9^^,^^13^

#### Hybrid features

The hybrid features include MEWS component scores (rather than the total MEWS score) and HPM-ExpertSignals features. Since the total MEWS score itself was used as a classifier, we chose component scores as clinical features without directly leveraging the output of MEWS. This approach provided an interpretable scheme to normalize the clinical values while preserving patterns across the feature set.

### Temporal clustering: k-shape

#### k-Shape

**Figure 2.**
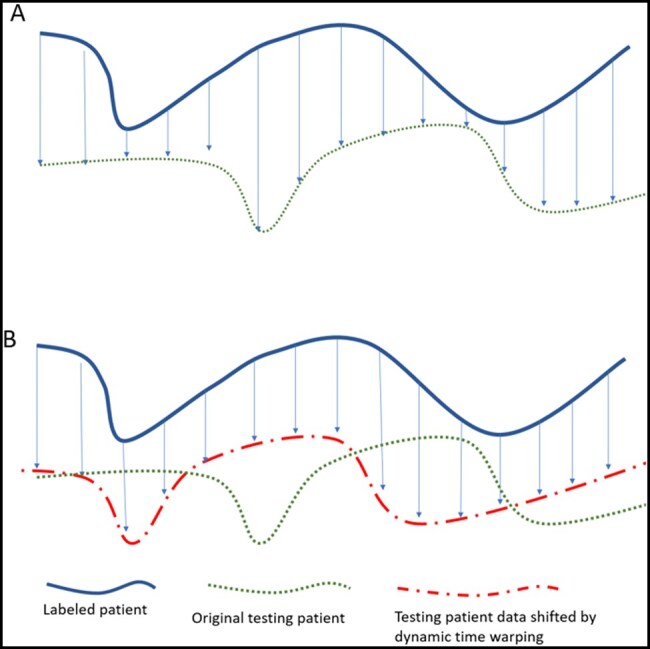
Contrasting 2 approaches for calculating the similarity between temporal patterns. (A) Algorithms that compare temporal patterns by matching fixed-hour intervals. (B) Temporal clustering techniques, such as k-Shape, calculate similarity by mathematically shifting patterns using dynamic time warping to find the optimal match.

Different temporal reasoning techniques vary in the types of information lost and signal gains. Basic techniques such as minimums, maximums, or averages over the time series are straightforward but do not capture more subtle temporal patterns (Figure 2A).^25^ More advanced methods that represent patterns using mathematical equations and coefficients assume the data shape remains consistent across fixed, predefined time intervals (eg, a sharp rise at the 10th hour followed by a slow decline at the 11th hour).^25^ Other sophisticated deep-learning techniques also rely on fixed-hour patterns.^25^ There are several ML techniques that are designed to handle temporal irregularly sampled data.^26^ For shape detection and to test our hypothesis, we have chosen k-Shape out of its simplicity and easy interpretation.

k-Shape is an unsupervised learning technique that uses multivariate temporal data as predictors.^15^ Its flexibility lies in employing dynamic time warping as a similarity function.^15^ Dynamic time warping calculates the distance between 2 temporal patterns by generating all possible alignment combinations and selecting the best-fit alignment (minimum distance) as the actual distance value (Figure 2B).^27^ This approach enables grouping similar patterns regardless of time gaps between offsets (eg, a sharp rise followed by a slow decline). Consequently, k-Shape may be more effective at capturing varying trends in clinical signs of deterioration and increased observation frequency. As an unsupervised ML technique, k-Shape has the potential to improve prediction performance by detecting discriminating signals even when the time gaps vary. The hourly sum of observation frequencies and the average MEWS scores per hour were prepared before clustering. For naïve models without temporal clustering, observation frequencies for each type within 24-h windows (from 36 to 12 h before events) were used as features.

### Models

We developed 6 models based on 3 feature combinations, each with or without (naïve) temporal clustering. As with prior studies^12^^,^^13^ each patient will have 1 ML model’s predictions 12 h before adverse events or discharge. The choice of the 12-h period prior to events or nonevents (discharge) allows us to evaluate the model’s predictive, not detection, performance with 1 standard snapshot per patient. However, in practice, models will make continuous predictions. The differences between the models are summarized in Table 1.

#### Modified early warning score

The MEWS was calculated using the last series of clinical observations recorded before the 12-h prediction window. A threshold score of 4 or above was selected to balance sensitivity and false positive rates, consistent with the use of MEWS in routine care.^23^^,^^28^

#### HMP-ExpertSignals (Naïve)

Observation frequencies for each type across the entire 24-h window (from 36 to 12 h before an event) were used as features to train an LR model, as the use of LR yielded favorable results previously.^13^

#### Hybrid-LR (Naïve)

The MEWS component scores from the last hour of the 24-h window and the total frequency of each observation type over the 24-h window were combined to develop an LR model.

#### MEWS with k-Shape

Time series of hourly MEWS component scores were clustered using k-Shape. The resulting cluster labels that produced best prediction performance were used as classifiers.

#### HPM-ExpertSignals with k-Shape

Time series of hourly observation frequencies were clustered using k-Shape. The resulting cluster labels that produced best prediction performance were used as classifiers.

#### Hybrid k-Shape

Modified early warning score and HPM-ExpertSignals time series were clustered using k-Shape, and the resulting cluster labels that had the best performance were used as classifiers.

### Study procedure

**Figure 3.**
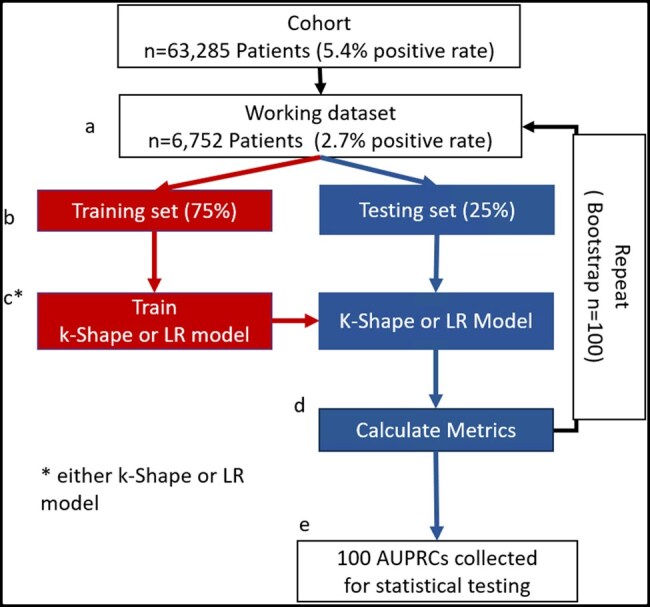
Study procedure. (a) A working dataset was sampled from the cohort. (b) The data were split into training and testing sets. (c) The training dataset was used to train an LR model or to create clusters using k-Shape. (d) Testing data were input into the pretrained models (LR or k-Shape). (e) The bootstrap process was repeated 100 times, and the resulting metrics were used for statistical hypothesis testing.

Performance measures were obtained through 100 bootstrap iterations. In each iteration, subsamples of patients were randomly drawn from the dataset to match the sample size and positive rates reported by Fu et al (Figure 3A).^13^ The subsamples were then randomly split into training and testing datasets (Figure 3B). For models without k-Shape, training data features were standardized and input into the LR model, with hyperparameters tuned using the training data. For k-Shape models, training data features were used to generate clusters (Figure 3C). The resulting cluster labels served as classifiers. The trained LR models or clusters were then used to classify the testing data. F-measures from the testing data were calculated for each of the 100 iterations (Figure 3D). Typically, to test the performance of a final model, data are split into train-validation-test splits with a single final hold-out cohort for model testing. However, since our objective was not to produce a final model, but to assess the statistical significance of performance differences across models, we repeated multiple train-test split cycles, training a new model instance in each, to generate a distribution for statistical analysis.

### Statistical analysis

The Friedman test assessed differences in F-measure across all 6 models. If the differences were significant at *α* = 0.05, 5 pairwise comparisons were performed using the Nemenyi post hoc test.^29^ Two pairwise comparisons were conducted to test the first hypothesis: (1) Hybrid-LR vs MEWS and (2) Hybrid-LR vs HPM-ExpertSignals. To evaluate the second hypothesis, 3 pairwise comparisons were performed: (1) MEWS vs MEWS with k-Shape, (2) HPM-ExpertSignals vs HPM-ExpertSignals with k-Shape, and (3) Hybrid vs Hybrid with k-Shape.

## Results

### The cohort’s dataset

The cohort included 63 285 patient admissions, of which 3420 (5.4%) experienced deterioration, comprising 3146 deaths (4.9%) and 274 cardiac arrests (0.4%) (Table 2). Positive cases had significantly more recorded observations than negative cases, except for nursing notes, which showed no significant difference.

**Table 2.** Characteristics of the study dataset.

Patients	Counts/Values	Positive cases	Negative cases	*P*-value
	63 285	3420 (5.4%)	59 865 (94.6%)	
Cardiac arrest *n* (%)		274 (0.4%)		
EXPIRED *n* (%)		3146 (4.9%)		
Age (years)	Range: 18-89	18-89	18-89	
	Mean: 63.2	66.1	62.8	<.001
Quartiles values (1st, 2nd, and 3rd IQR)	53.0, 65.0, 76.0	58.0, 68.0, 78.0	53.0, 65.0, 75.0	
Male, *n* (%)	34 458 (54.4%)	1909 (55.8%)	32 549 (54.4%)	
Number of observations, mean (SD)				
Heart rate	25.4 (20.9)	37.5 (29.6)	24.7 (20.1)	<.001
Respirator rate	22.8 (20.7)	33.5 (29.6)	22.2 (19.9)	<.001
Blood pressure	22.4 (18.4)	35.3 (29.0)	21.6 (17.3)	<.001
Body temperature	7.3 (9.5)	12.0 (17.7)	7.0 (8.8)	<.001
Oxygen saturation	21.5 (20.45)	30.8 (28.0)	21.0 (19.8)	<.001
Medication orders	3.2 (3.4)	5.2 (5.6)	3.2 (3.3)	<.001
Notes	1.3 (4.8)	1.3 (5.2)	1.3 (4.8)	.1

### Comparing Hybrid-LR with MEWS and HPM-ExpertSignals

Among the naïve models without temporal clustering, the Hybrid-LR model yielded a significantly higher F-measure than MEWS (0.25 [95% CI, 0.24-0.26] vs 0.18 [95% CI, 0.18-0.2]) and HPM-ExpertSignals alone (0.25 [95% CI, 0.24-0.26] vs 0.16 [95% CI, 0.15-0.17]) (Table 3).

**Table 3.** Primary and secondary outcomes comparing naïve models: MEWS vs Hybrid-LR and HPM-ExpertSignals vs Hybrid-LR.

	F-Measure	Sensitivity	PPV	Specificity	NPV	AUPRC	AUROC
MEWS	0.16 (0.15-0.17)	0.24 (0.23-0.25)	0.12(0.11-0.13)	0.95 (0.95-0.95)	0.98 (0.98-0.98)	0.1 (0.1-0.11)	0.67 (0.66-0.68)
HPM-ExpertSignals	0.19 (0.18-0.2)	0.23 (0.21-0.24)	0.19 (0.18-0.21)	0.97 (0.96-0.97)	0.98 (0.98-0.98)	0.1 (0.09-0.11)	0.67 (0.66-0.68)
Hybrid-LR	**0.25 (0.24-0.26)**	**0.28 (0.26-0.29)**	**0.26 (0.24-0.28)**	0.97 (0.97-0.98)	0.98 (0.98-0.98)	**0.15 (0.14-0.16)**	**0.71 (0.7-0.72)**

All measures are reported along with a 95% CI. Bolded values indicate statistically significant differences.

Abbreviations: HPM-ExpertSignals, Healthcare Process Model-ExpertSignals; LR, logistic regression; MEWS, modified early warning score; PPV, positive predictive value; NPV, negative predictive value.

The Hybrid-LR model outperformed MEWS and HPM-ExpertSignals in sensitivity (0.28 [0.26-0.29] vs 0.24 [0.23-0.25] vs 0.22 [0.21-0.24]), PPV (0.26 [0.24-0.28] vs 0.12 [0.11-0.13] vs 0.19 [0.18-0.21]), AUPRC (0.15 [0.14-0.16] vs 0.1 [0.1-0.11] vs 0.1 [0.09-0.11]), and AUROC (0.71 [0.7-0.72] vs 0.67 [0.66-0.68] vs 0.67 [0.66-0.68]). Specificity and negative predictive value (NPV) yielded no significant differences in the 2 comparisons (Table 3).

### Comparing models with and without k-Shape for each feature configuration

The 3 models using temporal clustering had significantly lower F-measure than any of the corresponding naïve models (MEWS + k-Shape 0.06 [95% CI, 0.06-0.06] vs MEWS 0.18 [95% CI, 0.18-0.2], HPM-Expert Signals + k-Shape 0.06 [95% CI, 0.06-0.06] vs HPM-Expert Signals 0.16 [95% CI, 0.15-0.17], Hybrid-LR + k-Shape 0.06 [95% CI, 0.06-0.06] vs Hybrid-LR 0.25 [95% CI, 0.24-0.26]). The k-Shape models had higher sensitivity but lower PPV and specificity than naïve models (Table 4). Purity of all k-Shape models was 0.97, with a positive rate of 3% in the testing population.

**Table 4.** Primary and secondary outcomes comparing k-Shape models with their corresponding naïve models.

	F-Measure	Sensitivity	PPV	Specificity	NPV	Purity score
MEWS	**0.16 (0.15-0.17)**	**0.24 (0.23-0.25)**	**0.12 (0.11-0.13)**	**0.95 (0.95-0.95)**	**0.98 (0.98-0.98)**	NA
MEWS with k-Shape	0.06 (0.06-0.06)	0.53 (0.51-0.55)	0.03 (0.03-0.03)	0.54(0.52-0.56)	0.98(0.98-0.98)	0.97 (0.97-0.97)
HPM-ExpertSignals	**0.19 (0.18-0.2)**	**0.23 (0.21-0.24)**	**0.19 (0.18-0.21)**	**0.97 (0.96-0.97)**	**0.98 (0.98-0.98)**	NA
HPM-ExpertSignals with k-Shape	0.06 (0.06-0.06)	0.58 (0.55-0.6)	0.03 (0.03-0.03)	0.49(0.47-0.52)	0.98 (0.98-0.98)	0.97 (0.97-0.97)
Hybrid-LR	**0.25 (0.24-0.26)**	**0.28 (0.26-0.29)**	**0.26 (0.24-0.28)**	**0.97 (0.97-0.98)**	**0.98 (0.98-0.98)**	NA
Hybrid-LR with k-Shape	0.06 (0.06-0.06)	0.58 (0.55-0.61)	0.03 (0.03-0.03)	0.48 (0.44-0.51)	0.97 (0.97-0.97)	0.27 (0.25-0.29)

All measures are reported along with a 95% CI. Bolded values indicate statistically significant differences within each pairwise comparison. Purity score only reported on k-Shape models, others were marked with “NA”.

Abbreviations: HPM-ExpertSignals, Healthcare Process Model-ExpertSignals; LR, logistic regression; MEWS, modified early warning score; PPV, positive predictive value; NPV, negative predictive value.

## Discussion

This study examined different feature types and temporal clustering for predicting in-hospital deterioration in ICUs. The findings supported the first hypothesis: hybrid models that leverage both clinical and clinician-behavioral features outperform models that use only 1 feature type. However, the findings did not support the second hypothesis, as naïve models consistently achieved significantly higher F-measures than k-Shape models across all feature types.

Using MEWS as a baseline, the hybrid model achieved 3 times higher PPV, which, in clinical practice, would reduce the number of false alarms by one-third. The results indicate that certain elements of “knowing the patient,” as shown through nurses’ documentation habits detected by HPM-ExpertSignal features, may at times offer greater insight than clinical data alone. Conversely, MEWS can identify certain at-risk patients overlooked by nurses. The improved performance indicates that clinical and nurse documentation may be synergistic.

A PPV of 0.03 for data samples with a positivity rate of 0.027, along with a purity score of 0.97, suggests that k-Shape failed to capture any meaningful signal of deterioration. Since most cases were negative, the purity scores were not higher than chance, which indicates the clusters were poorly formulated. One possible explanation is that the 2 types of observation patterns—at-risk patients and not-at-risk patients—do not have distinct temporal observational patterns beyond increased frequency. Another explanation could be k-Shape would require more data points for pattern detection. Other k-Shape studies utilized hundreds of data points per sample.^15^ In this study, the mean observation frequency for blood pressure, heart rate, and respiratory rate was less than 1 per hour (Table 2), with high SDs, indicating noisy temporal patterns within a limited data window. Longer data windows improve the signal-to-noise ratio, enabling k-Shape to capture signals more effectively.

### Strengths and limitations

This study is the first to directly quantify the potential performance gain by combining nurses’ behavioral and clinical data into a single ML model (LR). It is also the first to explore the feasibility of temporal clustering for this prediction problem. Using a large, diverse dataset from hospitals across the United States further enhances the generalizability of the findings.

The CONCERN team’s recent clinical trial demonstrated the potential impact on patient outcomes in a real-world setting if a well-trained combined model is implemented into a well-designed dashboard.^11^ However, the objective of this report was only to quantify the benefits of HPM-ExpertSignals features in ML models and to explore if the data has distinct shapes that k-shape can detect. Therefore, the limitations of this study are as follows. First, we compared only a simpler model based on HPM-ExpertSignals, rather than the latest CONCERN EWS used in the clinical trial. The data used in the CONCERN EWS, such as seasonality, day of week, and common vs uncommon times, were not available in our dataset due to the deidentification procedure, which converted absolute timestamps into time offsets from admission. Improved lead time, which is both a key strength of CONCERN EWS and a clinically important metric, was not evaluated. Second, we compared only MEWS. Other models include the acute physiology and chronic health evaluation, simplified acute physiology score, and sequential organ failure assessment. Future studies should include comparisons with these ICU scoring systems.^30^ Third, the study was confined to ICU settings, and the findings may not generalize to patient deterioration in other contexts, such as inpatient wards or emergency rooms. Fourth, the use of a clustering algorithm should lend better visualization of the data itself. However, a more in-depth analysis of clustering shape was not carried out. Finally, other models designed for irregular time series, such as deep learning and transformer models, could have yielded better results overall. Identifying a more optimal model was beyond the scope of this study.

## Conclusion

Combining clinical features from MEWS with nursing documentation frequency (HPM-ExpertSignal), a proxy for nurses’ concern about a patient, improves performance. However, as temporal clustering (k-Shape) models performed worse than naïve models, the benefits and best methods for leveraging temporal trends within HPM-ExpertSignals data remain inconclusive. Future research should investigate how HPM-ExpertSignals can enhance other widely used scoring systems in clinical settings beyond ICUs and evaluate the effectiveness of alternative temporal abstraction techniques.

## Supplementary Material

ooag077_Supplementary_Data

## Data Availability

Data used in this research can be accessed from the eICU Collaborative Research Database via https://eicu-crd.mit.edu/
