# Supplementary material for: Enhancing prediction of inpatient deterioration by combining clinical and nurse concern features, with or without temporal clustering
Source: JAMIA Open. 2026 May 20;9(3):ooag077. doi: 10.1093/jamiaopen/ooag077 (PMC13188989; doi:10.1093/jamiaopen/ooag077)
Supplement: ooag077_Supplementary_Data [file ooag077_supplementary_data.docx]

**Supplement 1**

**URLs for the source code and data.**

**Original eICU-CRD data can be found here:**

[**https://physionet.org/content/eicu-crd-demo/2.0.1/**](https://physionet.org/content/eicu-crd-demo/2.0.1/)

**GitHub page can be found here:**

[**https://github.com/9929105/mews-hpm-kshape/tree/main**](https://github.com/9929105/mews-hpm-kshape/tree/main)
